# Infection control challenges in setting up community isolation and treatment facilities for patients with coronavirus disease 2019 (COVID-19): Implementation of directly observed environmental disinfection

**DOI:** 10.1017/ice.2020.1355

**Published:** 2020-12-07

**Authors:** Shuk-Ching Wong, Ming Leung, Danny Wah-Kun Tong, Larry Lap-Yip Lee, Will Lok-Hang Leung, Frank Wan-Kin Chan, Jonathan Hon-Kwan Chen, Ivan Fan-Ngai Hung, Kwok-Yung Yuen, Deacons Tai-Kong Yeung, Kin-Lai Chung, Vincent Chi-Chung Cheng

**Affiliations:** 1 Infection Control Team, Queen Mary Hospital, Hong Kong West Cluster, Hong Kong Special Administrative Region, China; 2 Nursing Services Department, Cluster Services, Hospital Authority, Hong Kong Special Administrative Region, China; 3 Department of Accident and Emergency, Tin Shui Wai Hospital, New Territories West Cluster, Hong Kong Special Administrative Region, China; 4 Department of Family Medicine and Primary Health Care, Kowloon West Cluster, Hospital Authority, Hong Kong Special Administrative Region, China; 5 Cluster Services, Hospital Authority, Hong Kong Special Administrative Region, China; 6 Department of Microbiology, Queen Mary Hospital, Hong Kong Special Administrative Region, China; 7 Department of Medicine, Li Ka Shing Faculty of Medicine, The University of Hong Kong, Hong Kong Special Administrative Region, China; 8 Department of Microbiology, Li Ka Shing Faculty of Medicine, The University of Hong Kong, Hong Kong Special Administrative Region, China; 9 Quality & Safety Division, Hospital Authority, Hong Kong Special Administrative Region, China

## Abstract

**Background:**

Extensive environmental contamination by severe acute respiratory syndrome coronavirus 2 (SARS-CoV-2) has been reported in hospitals during the coronavirus disease 2019 (COVID-19) pandemic. We report our experience with the practice of directly observed environmental disinfection (DOED) in a community isolation facility (CIF) and a community treatment facility (CTF) in Hong Kong.

**Methods:**

The CIF, with 250 single-room bungalows in a holiday camp, opened on July 24, 2020, to receive step-down patients from hospitals. The CTF, with 500 beds in open cubicles inside a convention hall, was activated on August 1, 2020, to admit newly diagnosed COVID-19 patients from the community. Healthcare workers (HCWs) and cleaning staff received infection control training to reinforce donning and doffing of personal protective equipment and to understand the practice of DOED, in which the cleaning staff observed patient and staff activities and then performed environmental disinfection immediately thereafter. Supervisors also observed cleaning staff to ensure the quality of work. In the CTF, air and environmental samples were collected on days 7, 14, 21, and 28 for SARS-CoV-2 detection by RT-PCR. Patient compliance with mask wearing was also recorded.

**Results:**

Of 291 HCWs and 54 cleaning staff who managed 243 patients in the CIF and 674 patients in the CTF from July 24 to August 29, 2020, no one acquired COVID-19. All 24 air samples and 520 environmental samples collected in the patient area of the CTF were negative for SARS-CoV-2. Patient compliance with mask wearing was 100%.

**Conclusion:**

With appropriate infection control measures, zero environmental contamination and nosocomial transmission of SARS-CoV-2 to HCWs and cleaning staff was achieved.

Pandemic coronavirus disease 2019 (COVID-19), caused by severe acute respiratory syndrome coronavirus 2 (SARS-CoV-2), continues to spread globally, with 53.7 million cases and 1.3 million deaths as of November 15, 2020.^1^ The number of infections per million population may vary among different countries or regions, depending on the national healthcare policy regarding control of COVID-19. During the initial phase of the pandemic, in early March 2020, Sweden and the United Kingdom aimed to achieve herd immunity, which resulted in higher rates of viral infection, hospitalization, and mortality per million population.^2^ From late March to late May 2020 in the United States, lockdown measures were implemented to reduce community transmission of the SARS-CoV-2. Universal use of face masks in public was encouraged to prevent a postlockdown resurgence of COVID-19.^3^ In Hong Kong, a city with a population of 7.5 million, we adopted public health measures, such as hand hygiene, social distancing, and universal masking, in both the community and hospitals at the beginning of the COVID-19 era.^4-7^ In addition, we retained close contacts of COVID-19 cases in quarantine centers, and we contained all confirmed COVID-19 cases in the airborne infection isolation rooms (AIIRs) of hospitals. Infection control measures were enforced to minimize the risk of community and nosocomial transmission.^8,9^ However, these containment measures have imposed an increasing burden to the healthcare system, especially during the third wave of COVID-19.^10^ Therefore, we established a community isolation facility (CIF) to care for clinically stable patients stepping down from the AIIRs of hospitals as well as a community treatment facility (CTF) to house all newly diagnosed COVID-19 patients aged 18–60 years to prevent overcrowding in the hospitals.

We aimed to achieve zero nosocomial cases of COVID-19 among HCWs.^11^ Therefore, infection control measures were strategically important in the set-up of the CIF and CTF. In addition to the design of layout and workflow to minimize the risk of cross contamination, we introduced a novel concept of directly observed environmental disinfection (DOED) in our community facilities, and we achieved zero environmental contamination there. Our findings may facilitate the infection control preparedness of others facing the surge of COVID-19 as the pandemic progresses.

## Methods

### Setting up a community isolation facility (CIF) and a community treatment facility (CTF) for COVID-19 patients

As part of the infection control preparedness and responses to the COVID-19 pandemic, all confirmed cases have been cared for in AIIRs,^9^ and patients have been discharged to the community if they are clinical stable and fulfill either of the following laboratory criteria: (1) 2 clinical specimens of the same type (ie, respiratory or stool sample) tested negative for SARS-CoV-2 by reverse transcription polymerase chain reaction (RT-PCR) assay at least 24 hours apart or (2) a positive test for SARS-CoV-2 antibody, anti–receptor-binding domain of the viral spike protein (ie, anti-RBD IgG). As COVID-19 surveillance in both the hospital and community settings has been progressively enhanced, the CIF and CTF have been made available to relieve overcrowding in hospitals.

Planning for the CIF began in April 2020. It was set up at Lei Yue Mun Park and Holiday Village (known as LYM hereafter) and was activated on July 24, 2020 (day 207 after the official announcement of pneumonia outbreak in Wuhan, China, on December 31, 2019). With an area of 230,000 m^2^, LYM is located in the Chai Wan District on Hong Kong Island overlooking Lei Yue Mun Channel.^12^ Bungalows were temporarily built on 2 basketball courts to accommodate 120 and 250 patients in 2 wings at sites A and B, respectively. Each patient occupied a bungalow of 15 m^2^. Patients could be transferred from hospitals and stayed in the bungalows at the CIF if the step-down criteria were fulfilled (Table 1).

**Table 1.** Step-Down Criteria to Community Isolation Facility for Confirmed COVID-19 Patients^a,b^

Demographics^c^	Clinical and Laboratory^c^	Treatment^c^
Age <50 y^d^	Afebrile > 48 h	Not on antiviral therapy
ADL independent	No diarrhea	Not on oxygen therapy
No major comorbidity^e^	Normal or improving trend of hematology and biochemistry blood test profile^f^	

Note. ADL, activities of daily living.

a Community isolation facility at Lei Yue Mun Park and Holiday Village was activated on July 24, 2020 (day 207), to receive confirmed COVID-19 patients from the airborne infection isolation rooms (AIIRs) from hospitals.

b Cycle threshold value is a consideration factor but not a mandatory criteria for step-down to community isolation facility. If the patient deteriorates clinically, the patient will be transferred back to an AIIR in the hospital.

c Demographic, clinical and laboratory, and treatment criteria are all inclusive.

d Decision to transfer patients aged 50–60 y is subjected to case-by-case assessment by physician in-charge between hospitals and the community isolation facility.

e Such as diabetes mellitus, chronic pulmonary diseases, and immunocompromised conditions.

f Such as lymphocytes, lactate dehydrogenase, and C-reactive protein.

**Fig. 1.**
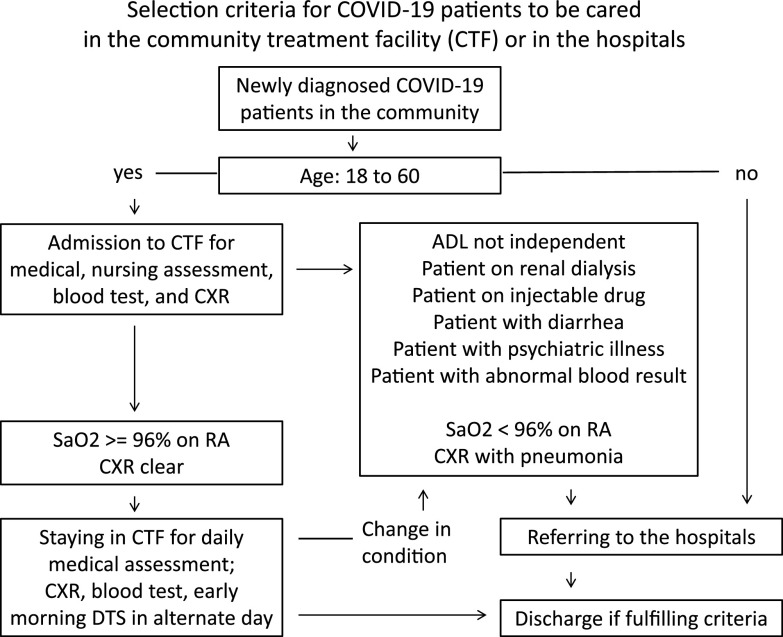
Selection criteria for COVID-19 patients to be cared in the community treatment facility or in the hospitals. Note. ADL, activities of daily living; CTF, community treatment facility; CXR, chest radiograph; DTS, deep throat saliva; RA, room air; SaO_2_, oxygen saturation. For the patient discharge criteria, patients can be discharged to the community if they are clinical stable and fulfill either of the following laboratory criteria: (1) with 2 clinical specimens of the same type (ie, respiratory or stool sample) tested negative for SARS-CoV-2 by reverse transcription polymerase chain reaction (RT-PCR) taken at least 24 hours apart, or (2) tested positive for SARS-CoV-2 antibody, anti–receptor-binding domain of the viral spike protein (anti-RBD IgG).

Planning for the CTF began in June 2020. It was established at the AsiaWorld-Expo (AWE) located adjacent to the Hong Kong International Airport and activated on August 1, 2020 (day 215). The AWE facility offers >70,000 m^2^ of space for exhibitions, concerts, and entertainment events.^13^ Two halls at the AWE, hall 1 (500 beds in 10,880 m^2^ and 19 m ceiling) and hall 2 (400 beds in 10,100 m^2^ and 10 m ceiling) were converted into open-cubicle bays. The CTF served as a triage center for newly diagnosed COVID-19 patients aged 18–60 years from the community according to our protocol (Fig. 1).

### Infection control logistics and measures in the CIF and CTF

In preparing and controlling the COVID-19 pandemic in our facilities, we aimed to achieve zero nosocomial infections among healthcare workers (HCWs).^11,14^ In the CIF and CTF, an infection control officer (ICO) and an infection control nurse (ICN) were appointed to be responsible for the design, coordination, supervision, and audit of the infection control measures to ensure staff safety. Both the CIF and CTF were designed with clear segregation between patient and staff areas. Right-on-time infection control sharing was provided to all HCWs and to cleaning and supporting staff working in the patient areas of the CIF and CTF. The HCWs and cleaning staff were required to seek medical attention and to be tested for SARS-CoV-2 if they had fever, respiratory, or diarrhea symptoms. Outbreak investigations were performed to determine the likelihood of nosocomial transmission of COVID-19. Contact between HCWs and patients was minimized by using telemedicine to facilitate clinical assessment and by introducing self-collection of deep throat saliva in the morning for virological monitoring. Universal masking of patients was adopted. Patient compliance with surgical masks was regularly monitored. The air ventilation system fulfilling the World Health organization (WHO) standard for healthcare facilities was installed;^15^ our ventilation flow rate was 80 L per second per person in the CTF. Without the installation of high-efficiency particulate air filter, fans were placed adjacent to the entrance and exit, directing airflow from staff area (green zone) to patient area (red zone).

### Directly observed environmental disinfection (DOED) in the CIF and CTF

To the CIF and CTF, we also introduced DOED. The 54 members of the cleaning staff were strategically placed in areas of the CIF and CTF to observe patient and staff activities. The cleaning staff performed environmental disinfection immediately after any activities. Supervisors of the cleaning team also observed cleaning staff directly or via closed-circuit television, and they reminded the staff directly or via walkie talkie (handheld transceiver) when necessary to ensure the quality of environmental disinfection. Supervisors conducted briefings and debriefings with the cleaning team before and after each work shift to reinforce DOED practices.

### Collection of air samples and environmental samples in the CTF for SARS-CoV-2 testing

Since patients received care in open cubicles and walked freely throughout the patient areas of the CTF, air and environmental samples were prospectively monitored for SARS-CoV-2 RNA on days 7, 14, 21, and 28 after the CTF opened. Air samples were collected in 6 different areas using a SAS Super ISO 180 model 86834 air sampler (VWR International PBI, Milan, Italy) as previously described.^9,16,17^ In addition, 3 air samples were collected at the bedsides of the 3 patients with highest viral loads among all patients on the day of air sample collection. Another 3 air samples were collected at the registration area, and 2 recreational areas in hall 1 of the AWE CTF. Briefly, the air sampler was perpendicularly positioned, and 1,000 L air at a rate of 180 L per minute was collected for each culture plate containing 3 mL viral transport medium (VTM). The VTM, kept at 4°C, was transferred to the laboratory within 4 hours and was subjected to RT-PCR assay for the detection of SARS-CoV-2.

Swab samples were collected from the surfaces of the patient registration area, the patient waiting area, the blood taking area, 10 vital-signs monitoring stations, 2 patient recreation areas, the radiograph waiting area, the radiograph machine, and 6 doffing areas. Swabs were then submerged in a tube containing 2 mL VTM. Each tube was spun in a vortexer and centrifuged at 13,000×*g* for 1 minute, then 1 mL supernatant was used for nucleic acid extraction and RT-PCR. Details of the laboratory SARS-CoV-2 testing procedures are provided in Supplementary File 1 (online).

### Statistical analysis

One sample t-test was used to compare the occupancy of the CTF at the days of samples collection with the average occupancy during the study period. A *P* value of <.05 was considered statistically significant.

## Results

### Setting up the CIF and CTF for COVID-19 patients

**Fig. 2.**
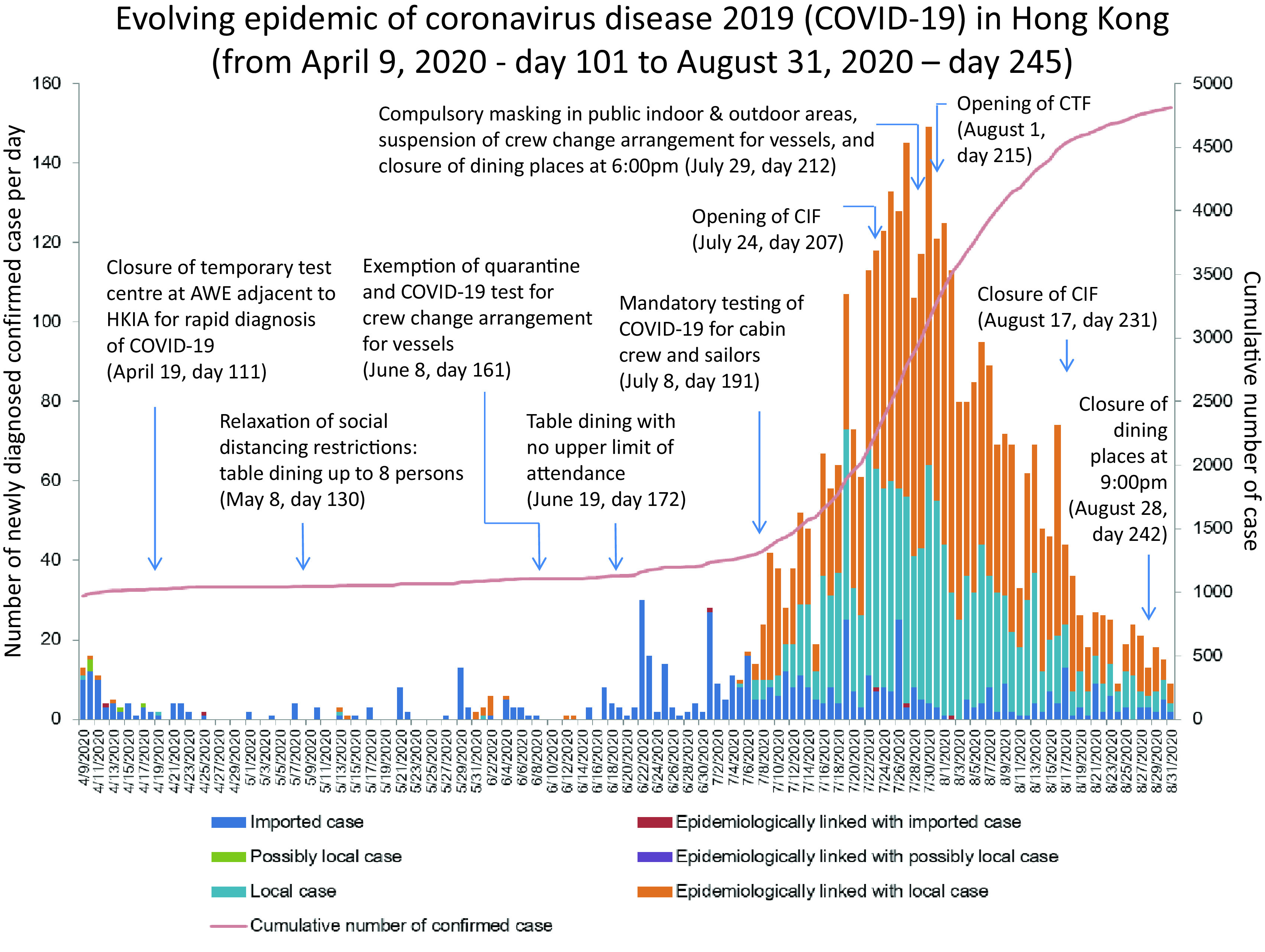
Evolving epidemic of COVID-19 in Hong Kong from April 9, 2020 (day 101) to August 31, 2020 (day 245). Official announcement of the community acquired pneumonia outbreak in Wuhan, Hubei Province by National Health Commission of the People’s Republic of China, on December 31, 2019 is defined as day 1. Note. AWE, AsiaWorld-Expo; HKIA, Hong Kong International Airport; CIF, community isolation facility at Lei Yue Mun Park and Holiday Village; CTF, community treatment facility at AsiaWorld-Expo adjacent to the Hong Kong International Airport.

**Fig. 3.**
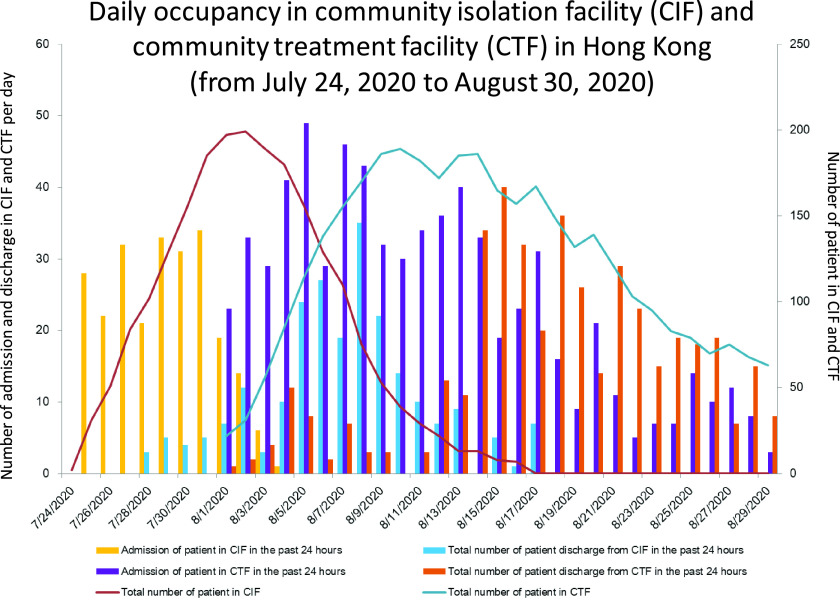
Daily occupancy in community isolation facility (CIF) and community treatment facility (CTF) in Hong Kong. In the CIF at Lei Yue Mun Park and Holiday Village, only site B was opened for step-down care of COVID-19 patients on July 24, 2020 (day 207). Site A was maintained in standby mode. Since the CTF at AsiaWorld-Expo adjacent to the Hong Kong International Airport opened on August 1, 2020 (day 215), and the community outbreak of COVID-19 was under control, the CIF was converted to standby mode on August 17, 2020 (day 231).

The third wave of COVID-19 began on July 5, 2020 (day 188), when the number of locally infected cases of unknown origin began to increase. The cumulative number of COVID-19 patients increased from 1,269 on day 188 to 2,373 on day 207 (July 24, 2020) (Fig. 2). This increase led to the activation of the first phase of the CIF with 250 single-room bungalows at site B of LYM in Hong Kong. The daily number of admissions and occupancy at the CIF are shown in Figure 3. In the CIF from July 24, 2020 (day 207), to August 17, 2020 (day 231), 243 patients (110 men and 133 women 133; median age, 35 years) were transferred from hospitals and subsequently discharged home. The median length of stay of a patient in the CIF was 9 days (range, 1–22).

The first phase of the CTF opened on August 1, 2020 (day 215) with 500 beds in open cubicles in hall 1 of the AWE. In total, 674 newly diagnosed COVID-19 patients were admitted to the CTF for triage in the first 4 weeks, 345 men and 329 women with a median age of 37 years (range, 7–60). During this time, 505 of 674 patients (74.9%) were discharged from the CTF. The median length of stay among patients in the CTF was 4 days (range, 1–19). Overall, 138 of 674 patients (20.5%) were transferred to hospitals according to our protocol (Fig. 1).

### Infection control logistics and measures in the CIF and CTF

The HCWs were recruited from 7 healthcare networks under the governance of the Hospital Authority. Six sessions of infection control training were arranged for 67 doctors, 120 nurses, 69 supporting staff, 22 pharmacists, and 13 radiographers to explain the workflow and to reinforce infection control measures in the CIF and CTF. Demonstrations of donning and doffing of personal protective equipment (PPE) were performed, and the HCWs were audited for the appropriate donning and doffing of PPE. Directly observed donning and doffing of PPE was practiced as previously described.^18^

**Fig. 4.**
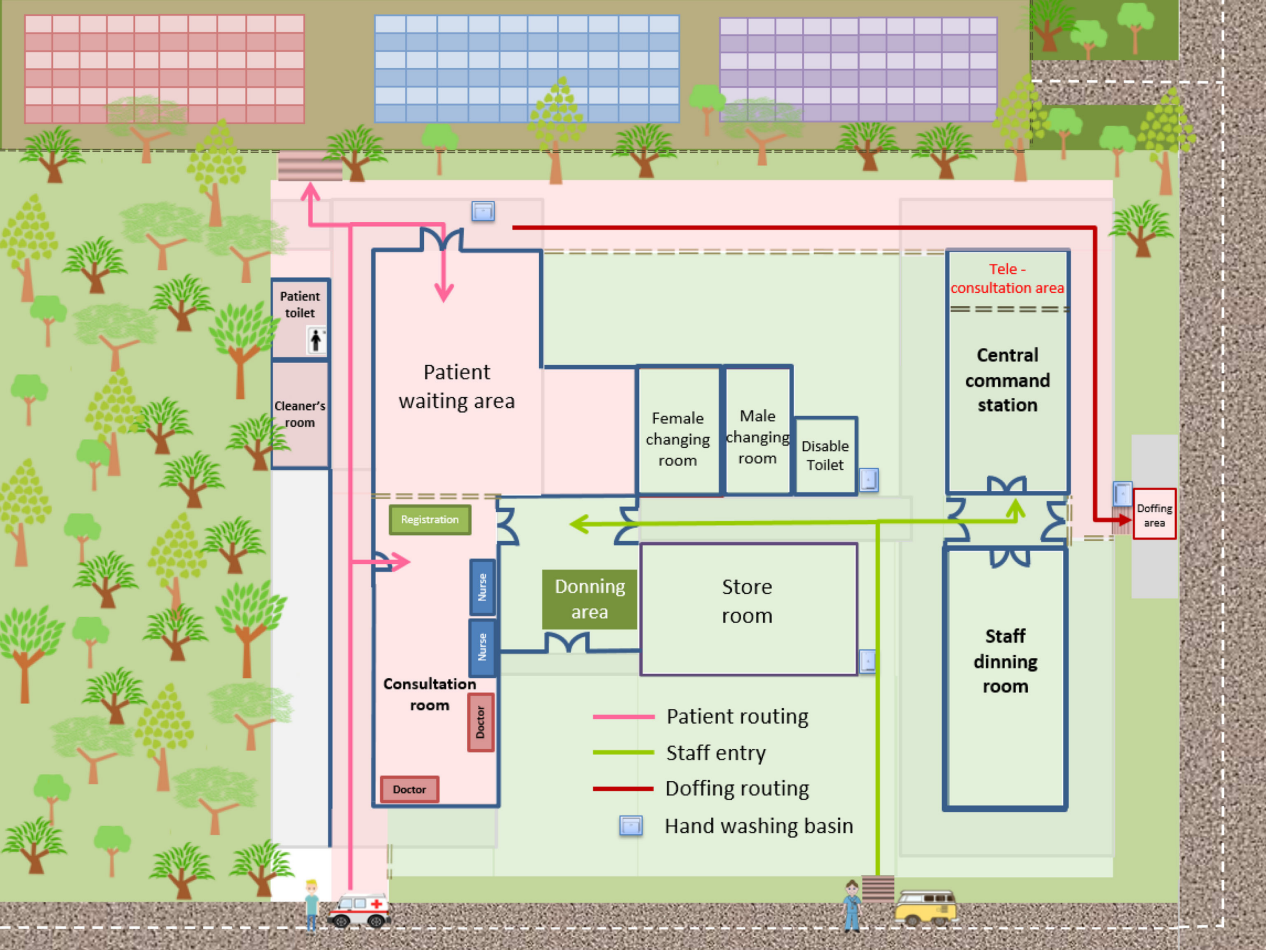
Layout and workflow of the community isolation facility (CIF) at Lei Yue Mun Park and Holiday Village. Note. Patient area (red zone) and staff area (green zone) are marked in red and green as the background colors, respectively. Healthcare workers (HCWs) were assigned a designated entrance and route to the central command station to report duty during the day or night shift. HCWs wore full personal protective equipment (PPE) with surgical respirator (ie, N95 respirator), cap, face shield, isolation gown, and gloves in the donning area before entering the consultation room. Upon ambulance arrival, HCWs with full PPE went out from the consultation room to escort patients from the ambulance drop-off point to the consultation room, where registration and clinical assessment were performed. Subsequently, patients were escorted to the patient waiting area to receive basic infection control training with emphasis on hand hygiene and wearing surgical mask, and to learn the housekeeping activities such as self-collection of deep throat saliva in the early morning, self-monitoring of blood pressure, pulse, and body temperature, the using of teleconsultation system inside patient rooms, as well as the delivery of meals and linens and disposal of wastes from the bungalows. HCWs then escorted patients to the assigned bungalow. Each bungalow occupied by either 1 patient or 1 family of 2 persons. HCWs returned from the bungalows, following a designated route for doffing and went to the central command station or staff dining room.

**Fig. 5.**
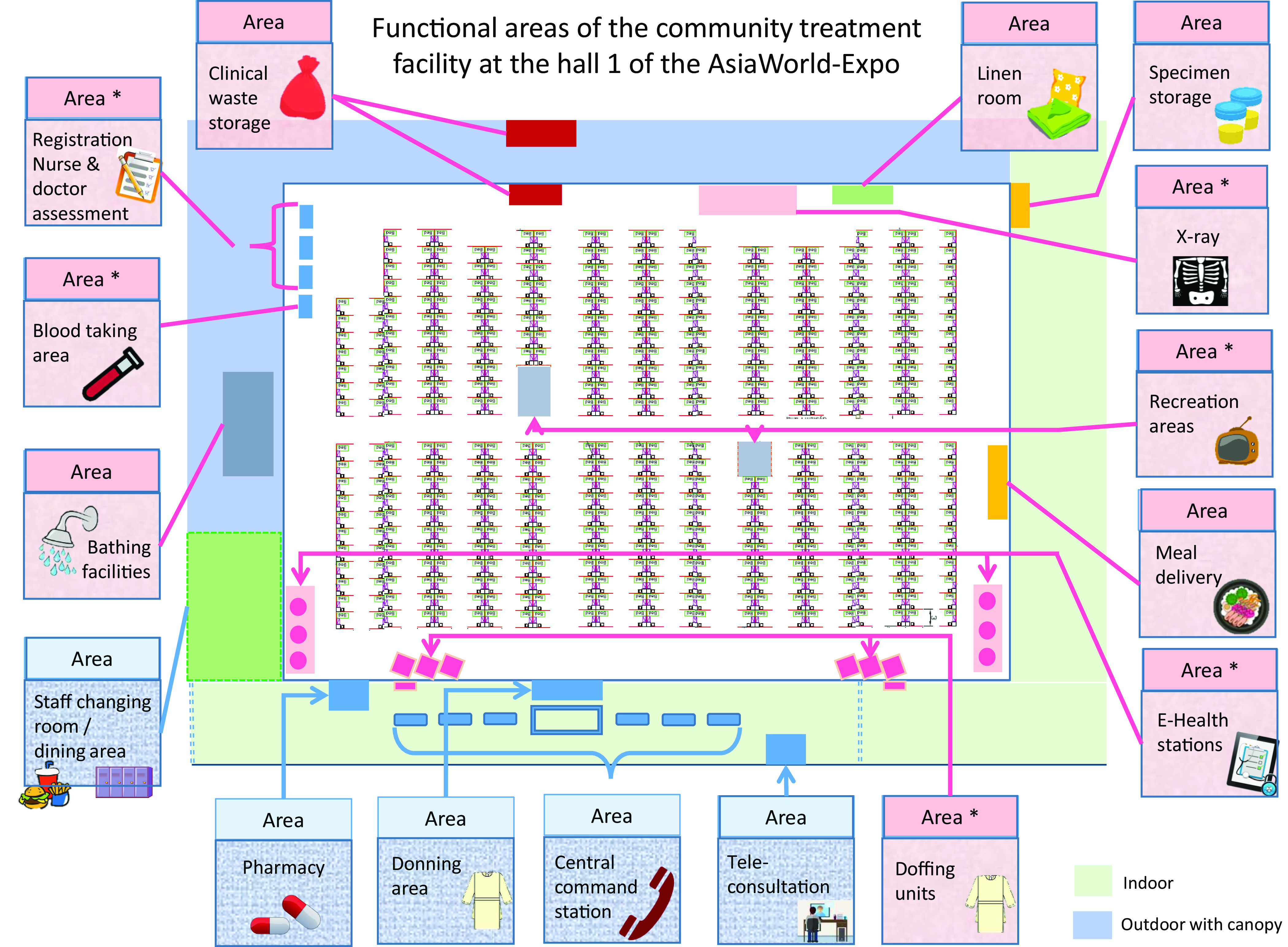
Functional areas of the community treatment facility (CTF) at the hall 1 of the AsiaWorld-Expo. Note. In the red zone, the functional areas marked with asterisk (*) represent the areas in frequent contact with either patients or healthcare workers. Environmental surface swabs were prospectively collected for 4 weeks to detect SARS-CoV-2 by RT-PCR. Air samples were collected at the registration area, 2 recreation areas, and at the bedsides of 3 patients with lowest, second lowest, and third lowest cycle threshold values of the deep throat saliva by RT-PCR on the day of sample collection. The registration area for nurse and doctor assessment, waiting area, blood taking area, radiograph area, E-health station, recreation area, and doffing area were disinfected manually by sodium hypochlorite solution (1,000 ppm) soaked in disposal wipe immediately after use under the scheme of directly observed environmental disinfection. If blood, secretions, vomitus, or excreta was spilled, the environmental surface was disinfected with sodium hypochlorite solution (10,000 ppm) soaked with a disposal wipe for 10 minutes before rinsing with water. Because the ventilation flow rate was 80 L per second per person and all patients wore surgical masks, it was not necessary to move the patients out during environmental disinfection.

The infection control logistics in the CIF relied on the segregation of patient and HCW routing. The patient area (red zone) and the staff area (green zone) were clearly delineated (Fig. 4). In the CTF, the exhibition hall was used for the patient area (red zone) where HCWs were required to wear appropriate PPE (ie, surgical respirator, cap, face shield, isolation gown, and gloves) in a 4-hour shift. The outside corridor was used for the staff area (green zone) where the central command center was set up. The layout, workflow, and functional areas at the CTF are shown in Supplementary Figure 1 (online) and Figure 5.

To minimize the contact between HCWs and patients in the CIF and CTF, blood pressure, body temperature, and oxygen saturation were self-monitored by patients using the electronic system, and the results were connected via Wi-Fi to the central monitoring system in the command center. Teleconsultation was conducted by medical and nursing staff daily and when clinically indicated. Deep throat saliva samples were collected by patients every morning, and anti-RBD IgG tests were performed as appropriate.

In the CIF and CTF, patients were also educated upon admission to reinforce infection control measures such as universal masking and practicing hand hygiene, especially before meal and medication,^19^ as well as before and after using the self-service vital-sign monitoring system. Pocket-sized bottles (60 mL) of alcohol-based hand rub were given to patients and large counter-top bottles (500 mL) were made available in the commonly shared areas. One surgical mask was given to each patient daily and upon request. Surgical mask compliance among patients in the CTF was 100% on day 7 after the CTF opened (170 of 170 patients), on day 14, (165 of 165 patients), on day 21 (103 of 103 patients), and on day 28 (63 of 63 patients). A window-based air conditioning system was installed in the bungalow of the CIF, and the air ventilation in the patient area of the CTF was equivalent to 80 L per second per person. Zero nosocomial COVID-19 was reported among HCWs and cleaning staff who worked in the CIF and the CTF.

### DOED in the CIF and CTF

Designated teams of 20 and 34 cleaning staff worked in the CIF and the CTF, respectively. The environment of commonly shared areas consisted of hard and nonporous surfaces: the patient registration area, the waiting area, the blood collection area, the radiograph area, the E-health station, the recreation area, and the doffing area. Immediately after use, these areas were disinfected manually using sodium hypochlorite solution (1,000 ppm) soaked in a disposal wipe under the DOED protocol. Terminal disinfection was performed in patient rooms in the CIF and in patient beds in the CTF. The green zone was disinfected using sodium hypochlorite solution (1,000 ppm) every 2 hours.

### Collection of air samples and environmental samples in the CTF for SARS-CoV-2 testing

Of 24 air samples and 520 environmental samples prospectively collected at weekly intervals for 4 weeks after the opening of the CTF, 4 of 24 air samples (16.7%) and 120 of 520 environmental surface samples (23.1%) were collected from the areas where HCWs had closely interacted with patients and areas used solely by HCWs. SARS-CoV-2 RNA was not detectable in any air or environmental sample by RT-PCR (Table 2). The occupancy of the CTF at the days of samples collection was not statistically difference as compared with the average occupancy during the study period (p = 0.95).

**Table 2.** Result of Air and Environmental Samples in Community Treatment Facility (CTF)^a^

Variable	Day After Opening of the CTF			
	Day 7^b^	Day 14^c^	Day 21^d^	Day 28^e^
Total no. of air samples collected	6	6	6	6
Total no. of environmental samples collected	130	130	130	130
Sites or items (number of sampling sites)				
**Air samples**				
Patient A (n=1)^f^	ND	ND	ND	ND
Patient B (n=1)^g^	ND	ND	ND	ND
Patient C (n=1)^h^	ND	ND	ND	ND
Patient registration area (n=1)^i^	ND	ND	ND	ND
Patient recreation area				
Recreation area A (n=1)	ND	ND	ND	ND
Recreation area B (n=1)	ND	ND	ND	ND
**Environmental samples**				
Patient registration area				
Table (n=7)	ND	ND	ND	ND
Chair (n=7)	ND	ND	ND	ND
Patient waiting area for registration				
Table (n=1)	ND	ND	ND	ND
Chair (n=12)	ND	ND	ND	ND
Blood taking area				
Table (n=4)	ND	ND	ND	ND
Chair (n=4)	ND	ND	ND	ND
Vital sign monitoring areas				
Table (n=10)	ND	ND	ND	ND
Chair (n=10)	ND	ND	ND	ND
Blood-pressure machine (n=10)	ND	ND	ND	ND
Electronic panel (n=10)	ND	ND	ND	ND
Patient recreation areas				
Table (n=2)	ND	ND	ND	ND
Chair (n=12)	ND	ND	ND	ND
Radiograph area				
Radiograph machine (n=1)	ND	ND	ND	ND
Chair (n=10)	ND	ND	ND	ND
Doffing areas for staff				
Table (n=6)	ND	ND	ND	ND
Mirror (n=6)	ND	ND	ND	ND
Partition (n=24)^j^	ND	ND	ND	ND

Note. ND, SARS-CoV-2 RNA was not detectable in the air or environmental samples.

a The CTF opened on August 1, 2020 (day 215). Hall 1 had 500 beds arranged in open cubicles, and hall 2 had 400 beds on standby mode. Air and environmental samples were collected in hall 1.

b In total, 170 patients (94 men and 76 women) were present when the air and environmental samples were collected.

c In total, 165 patients (85 men and 80 women) were present when the air and environmental samples were collected.

d In total, 103 patients (59 men and 44 women) were present when the air and environmental samples were collected.

e In total, 63 patients (34 men and 29 women) were present when the air and environmental samples were collected.

f Patient A represented the selected patient with lowest cycle threshold (Ct) value of the deep throat saliva by reverse transcription polymerase chain reaction (RT-PCR) testing on the day of air sampling. The Ct values of patient A in day 7, 14, 21, and 28 were 18.68, 17.61, 23.31, and 21.83, respectively.

g Patient B represented the selected patient with second lowest Ct value of the deep throat saliva by RT-PCR assay on the day of air sampling. The Ct values for patient B on day 7, 14, 21, and 28 were 23.65, 20.31, 23.48, and 28.54, respectively.

h Patient C represented the selected patient with third lowest Ct value of the deep throat saliva by RT-PCR assay on the day of air sampling. The Ct values for patient C on days 7, 14, 21, and 28 were 23.79, 23.55, 25.98, and 28.88, respectively.

i The area where the healthcare workers closely interacted with patients.

j Four partitions were used to build a single unit for removal of personal protective equipment. There were 6 units in the doffing areas, resulting in 24 partitions.

## Discussion

With the emergence of COVID-19 outbreaks in the community, we have been facing overwhelming challenges to our healthcare system. In addition to our recent experience setting up the temporary test center for COVID-19,^18^ we built a CIF and a CTF within 2 months. From planning to commissioning, we faced numerous hurdles. The CIF site was located in historical buildings under regulation of “Cap. 53 Antiquities and Monuments Ordinance” of Hong Kong,^20^ so modification of building structure was not allowed. However, we meticulously designed the workflow and patient and staff routing to minimize the risk of cross contamination according to the existing infrastructure, and we built the bungalows as single-room patient isolation units (Fig. 4). The CTF was located in a conventional hall, similar to the setting of temporary hospitals in Wuhan,^21,22^ the United States,^23^ the United Kingdom,^24,25^ and Singapore.^26^ We delineated a patient area (red zone) inside the hall and a staff area (green zone) in the corridor. A yellow zone was assigned for patient discharges and transfer of materials.

The CIF received step-down patients from the hospitals, and the CTF admitted newly diagnosed COVID-19 patients from the community. The SARS-CoV-2 viral load tends to be higher at the time of diagnosis;^27^ thus, the risk of environmental contamination may be higher. In fact, extensive contamination by SARS-CoV-2 have been reported in AIIRs^28^ as well as healthcare premises.^29^ Our previous study demonstrated that patients with viral loads of ≥3 log copies/mL in the clinical specimen were associated with environmental contamination.^30^ Therefore, we introduced a novel concept of DOED to ensure the environmental cleanliness in the CIF and CTF. We collected air and environmental surface samples in the commonly shared areas of the CTF where patients freely walked and exercised in the red zone. In our prospective weekly collection of samples, all 24 air samples and 520 environmental sites were negative for SARS-CoV-2 by RT-PCR. The negative results of air samples were consistent with our previous experiments conducted in AIIRs of different hospitals in Hong Kong.^9,30^ Although the air ventilation in the CTF was not based on the number of air changes per hour (ACH), it provided at least 80 L per second per person, which is equivalent to >6 ACH and complied with the WHO standard.^15^

For the environmental samples, we achieved zero contamination in the commonly shared areas used by patients (ie, registration, waiting, recreation, E-health, and radiograph areas) and in the doffing area used by HCWs for 4 consecutive weeks. The DOED protocol minimized the risk of environmental contamination, which has been shown to be a risk factor for nosocomial acquisition of multidrug-resistant organisms^31^ and SARS.^32^ Although the relationship between environmental contamination and acquisition of SARS-CoV-2 remains uncertain, DOED as a component of infection control measures deserves further investigation.

Universal masking of all patients in the CTF may prevent the dispersal of SARS-CoV-2 droplets and further reduce the risk of environmental contamination. During the 3-hour collection of air and environmental samples per session for 4 consecutive weeks, we monitored masking compliance, which was 100%. However, universal masking alone in the hospital does not achieve zero environmental contamination by SARS-CoV-2.^30^ We believe that DOED contributed to the achievement of zero environmental contamination in our CTF.

Education of HCWs was of utmost importance in achieving zero nosocomial transmission of COVID-19. All recruited HCWs were gathered for a half-day training session to introduce the workflow and infection control procedures with particular emphasis of directly observed donning and doffing of PPE, as previously described.^18^ The target of 100% hand hygiene compliance during COVID-19 era was shared with our HCWs.^33^

Since the CIF and CTF were outside the hospital premises, collaboration among different departments from the Hong Kong government was needed. Staff who worked at the CIF or CTF were offered infection control training by their ICO and ICN to ensure their physical and psychological preparedness. Timely support to these staff was crucial to the success of the CIF and CTF. For instance, during 2 episodes of drain blockage in a patient toilet in the CTF, the ICO and ICN of the CTF accompanied the facility management team with full PPE to observe the repair. The ICN also instructed and assisted the staff in doffing to ensure staff safety.

Zero nosocomial transmission of COVID-19 among HCWs remains our mission in the COVID-19 era.^11^ We aim to maintain this record among HCWs working in our hospitals, the CIF, and the CTF. We believe that our meticulous infection control measures with a combination of staff training, audit, promotion of staff and patient hand hygiene, direct observation of donning and doffing, as well as DOED are the keys to our success.

With the alleviation of SARS-CoV-2 transmission in the community, the CIF was temporarily closed and the number of patients also decreased in the CTF. However, the establishment of the CIF and the CTF serves as a model for combating any emerging infectious diseases to prevent hospital overcrowding. DOED is labor intensive, which may not be suitable for implementation in other healthcare settings. However, we believe our experience may be useful to others in the global response to COVID-19.

## Supplementary material

For supplementary material accompanying this paper visit https://doi.org/10.1017/ice.2020.1355. click here to view supplementary material
