# Supplementary material for: Infection control challenges in setting up community isolation and treatment facilities for patients with coronavirus disease 2019 (COVID-19): Implementation of directly observed environmental disinfection
Source: Infect Control Hosp Epidemiol. 2020 Dec 7:1–9. doi: 10.1017/ice.2020.1355 (PMC7889843; doi:10.1017/ice.2020.1355)
Supplement: Supplementary file 1 [file S0899823X20013550sup.zip › S0899823X20013550sup001.docx]

**Supplementary File**

***Laboratory diagnosis for SARS-CoV-2***

The patients’ early morning deep throat saliva, air samples and environmental swabs collected in CIF and CTF were subjected to nucleic acid extraction by the eMAG extraction system (bioMérieux, Marcy-l’Étoile France) as previously described.^1^ The presence of the SARS-CoV-2 RNA in the specimens was determined by the LightMix SarbecoV E-gene plus EAV control kit (TIB Molbiol, Berlin, Germany) according to the manufacturer’s instructions.^2,3^ The assay included an EAV extraction control which could monitor the presence of amplification inhibitors in the specimens to exclude a false negative result.

The SARS-CoV-2 IgG serology testing was performed on patients in CIF and CTF by using the FDA emergency use approved Abbott ARCHITECT SARS-CoV-2 IgG qualitative assay according to the manufacturer’s instructions.^4^ Briefly, 100 μl of patient’s serum was used for detection of the IgG antibodies targeting the SARS-CoV-2 nucleocapsid. Results were reported as an index (ratio of the chemiluminescent signal between the samples and a calibrator), with values >1.4 indicating a positive result.

References of Supplementary File

1. Chan JF, Chan KH, Choi GK, *et al.* Differential cell line susceptibility to the emerging novel human betacoronavirus 2c EMC/2012: implications for disease pathogenesis and clinical manifestation. *J Infect Dis* 2013;207:1743-52.
2. Corman VM, Landt O, Kaiser M, *et al.* Detection of 2019 novel coronavirus (2019-nCoV) by real-time RT-PCR. *Euro Surveill* 2020;25(3). Epub 2020/01/30. doi: 10.2807/1560-7917.ES.2020.25.3.2000045.
3. Yip CC, Sridhar S, Cheng AK, *et al.* Evaluation of the commercially available LightMix(R) Modular E-gene kit using clinical and proficiency testing specimens for SARS-CoV-2 detection. *J Clin Virol* 2020;129:104476. Epub 2020/06/10. doi: 10.1016/j.jcv.2020.104476.
4. Bryan A, Pepper G, Wener MH, *et al.* Performance Characteristics of the Abbott Architect SARS-CoV-2 IgG Assay and Seroprevalence in Boise, Idaho. *J Clin Microbiol* 2020;58(8). Epub 2020/05/10. doi: 10.1128/JCM.00941-20.
