# Supplementary material for: Infection control challenges in setting up community isolation and treatment facilities for patients with coronavirus disease 2019 (COVID-19): Implementation of directly observed environmental disinfection
Source: Infect Control Hosp Epidemiol. 2020 Dec 7:1–9. doi: 10.1017/ice.2020.1355 (PMC7889843; doi:10.1017/ice.2020.1355)
Supplement: Supplementary file 1 [file S0899823X20013550sup.zip › S0899823X20013550sup002.docx]

**Supplementary Figure**

**Layout and workflow of the community treatment facility at the Hall 1 of AsiaWorld-Expo**


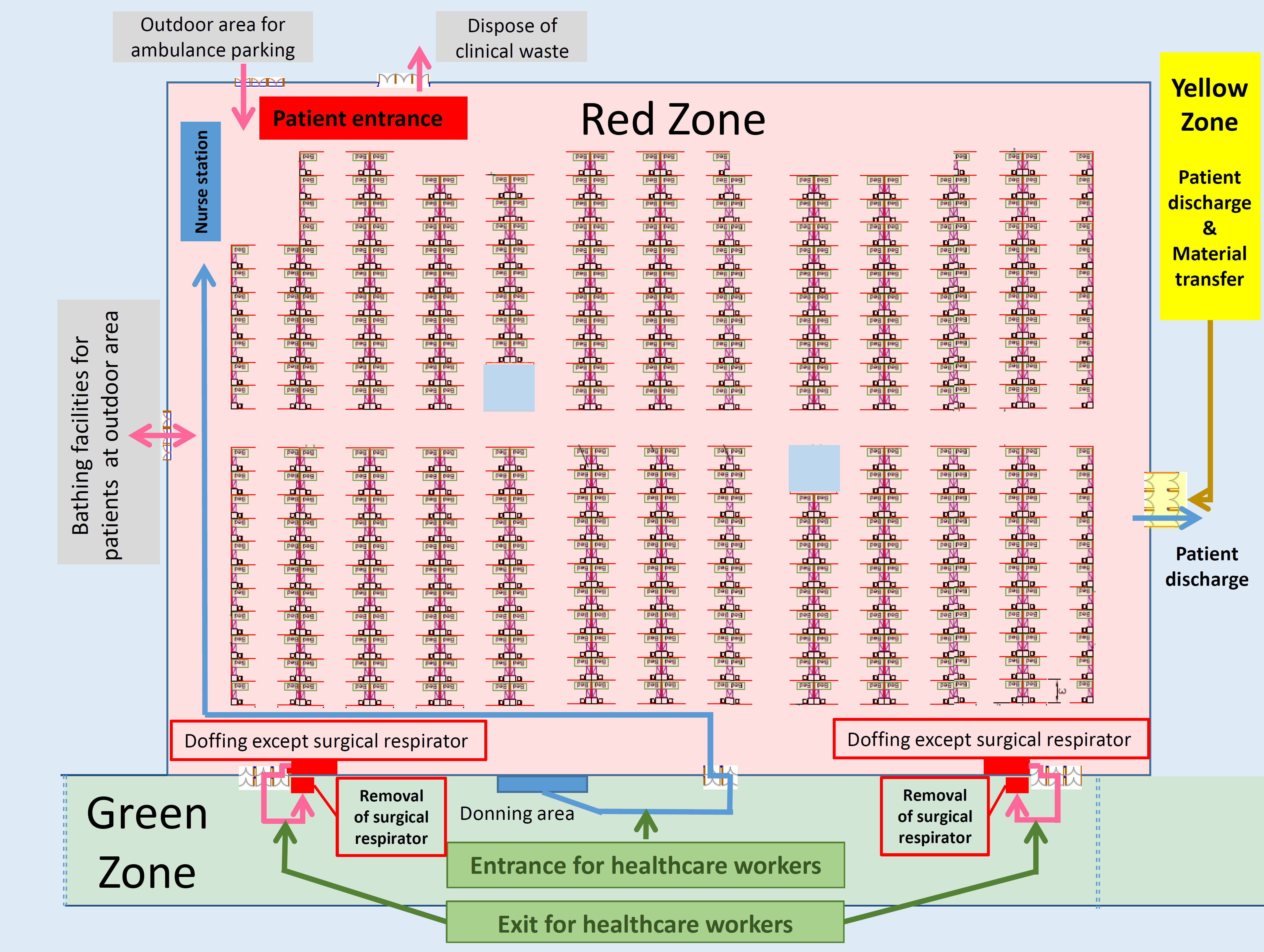


Note. The layout of Hall 1 is clearly segregated into staff area (green zone), which is the corridor of the hall, and patient area (red zone). Healthcare workers (HCWs) wore full personal protective equipment (PPE) with surgical respirator (i.e. N95 respirator), faceshield, isolation gown, and gloves in the donning area at green zone under direct observation by HCWs in the central commend station before entering the red zone. Newly diagnosed COVID-19 patients were transferred to CTF by ambulance, and reported to nurse station, which was placed next to the patient entrance. Registration and clinical assessment were performed. Patients received basic infection control training with emphasis of hand hygiene before and after meal and medication rounds, and use of E-health station, as well as wearing surgical mask all the time except bathing and sleeping. Patients were also reminded the housekeeping activities such as self-collection of deep throat saliva in the early morning, self-monitor of blood pressure, pulse, and temperature in the E-health station, the use of tele-consultation system, as well as the self-collection of meal. The bed linens were changed by patients themselves during their stay. The bathing facilities were located at the outdoor area with roof cover. A designated exit route was assigned for disposal of clinical waste from red zone. For patients with clinical deterioration requiring hospitalization, they would go through the reverse path of patient entrance route and pick up by ambulance. Two doffing areas were set in red zone. HCWs removed gloves, isolation gown, faceshield, and cap in sequence. Another two doffing areas were set in green zone for removal of surgical respirator. A yellow zone was assigned for discharging patients to the community and transferring materials such as meal and linens into the red zone.
